# Effects of Training and Competition Load on Neuromuscular Recovery, Testosterone, Cortisol, and Match Performance During a Season of Professional Football

**DOI:** 10.3389/fphys.2018.00668

**Published:** 2018-06-07

**Authors:** Amber E. Rowell, Robert J. Aughey, William G. Hopkins, Alizera Esmaeili, Brendan H. Lazarus, Stuart J. Cormack

**Affiliations:** ^1^Institute of Sport, Exercise and Active Living, Victoria University, Melbourne, VIC, Australia; ^2^Seattle Sounders Football Club, Seattle, WA, United States; ^3^Norwegian Institute for Defence Studies, Oslo, Norway; ^4^Collingwood Football Club, Melbourne, VIC, Australia; ^5^School of Exercise Science, Australian Catholic University, Melbourne, VIC, Australia

**Keywords:** team sport performance, mixed modeling, countermovement jump, salivary hormones, load monitoring

## Abstract

**Introduction:** Training load and other measures potentially related to match performance are routinely monitored in team-sport athletes. The aim of this research was to examine the effect of training load on such measures and on match performance during a season of professional football.

**Materials and Methods:** Training load was measured daily as session duration times perceived exertion in 23 A-League football players. Measures of exponentially weighted cumulative training load were calculated using decay factors representing time constants of 3–28 days. Players performed a countermovement jump for estimation of a measure of neuromuscular recovery (ratio of flight time to contraction time, FT:CT), and provided a saliva sample for measurement of testosterone and cortisol concentrations 1-day prior to each of 34 matches. Match performance was assessed via ratings provided by five coaching and fitness staff on a 5-point Likert scale. Effects of training load on FT:CT, hormone concentrations and match performance were modeled as quadratic predictors and expressed as changes in the outcome measure for a change in the predictor of one within-player standard deviation (1 SD) below and above the mean. Changes in each of five playing positions were assessed using standardization and magnitude-based inference.

**Results:** The largest effects of training were generally observed in the 3- to 14-day windows. Center defenders showed a small reduction in coach rating when 14-day a smoothed load increased from −1 SD to the mean (-0.31, ±0.15; mean, ±90% confidence limits), whereas strikers and wide midfielders displayed a small increase in coach rating when load increased 1 SD above the mean. The effects of training load on FT:CT were mostly unclear or trivial, but effects of training load on hormones included a large increase in cortisol (102, ±58%) and moderate increase in testosterone (24, ±18%) in center defenders when 3-day smoothed training load increased 1 SD above the mean. A 1 SD increase in training load above the mean generally resulted in substantial reductions in testosterone:cortisol ratio.

**Conclusion:** The effects of recent training on match performance and hormones in A-League football players highlight the importance of position-specific monitoring and training.

## Introduction

Maximizing performance and minimizing injury risk in team sport athletes requires a careful balance of applying training load and recovery (Borresen and Lambert, 2009; Halson, 2014; Jaspers et al., 2016). The application of a training stimulus has both positive (fitness) and negative (fatigue) outcomes (Foster, 1998; Smith, 2003; Drew and Finch, 2016). Performance is thus considered the function of fitness and fatigue. Whilst fitness is relatively slow to develop and decay, fatigue accumulates and dissipates more quickly (Smith, 2003; Murray et al., 2016; Williams et al., 2016b). Despite this high level knowledge, very little is known on the specific interactions between training load, the resultant fatigue response and subsequent performance (Aughey et al., 2016).

A common method of assessing internal load in team sports is via collection of the athlete rating of perceived exertion (RPE) (Impellizzeri et al., 2004; Borresen and Lambert, 2008; Halson, 2014) which is then multiplied by session duration to represent the internal load (session rating of perceived exertion, sRPE) (Foster, 1998; Foster et al., 2001; Scott et al., 2013). Chronic load (average sRPE over relatively longer periods of training, e.g., 4 weeks) has been suggested to represent fitness whilst acute load (sRPE over shorter periods, e.g., 1 week) may represent fatigue (Drew and Finch, 2016). Interestingly, high acute load is suggested to enhance performance (Sampson et al., 2016). Although training load for a given period is commonly calculated using rolling averages, this does not emphasize the likely greater importance of recent load (Murray et al., 2016; Sampson et al., 2016). Given this limitation, it has been suggested that an exponentially weighted moving average (EWMA) where different decay constants are applied to different length periods should be utilized (Murray et al., 2016; Williams et al., 2016a). Regardless of calculation method, it is unclear whether higher or lower training load is positively or negatively associated with football performance and whether this association varies across playing positions.

Whilst the response to match play in different sports has been assessed in various ways including measurement of hormones such as testosterone and cortisol (Cormack et al., 2008a,b), a recent investigation in elite football failed to show a clear dose–response relationship (Rowell et al., 2017). However, the impact of changes in the hormonal profile on subsequent match performance was not examined. In contrast, the ratio of flight time to contraction time (FT:CT) obtained from a countermovement jump (CMJ) is a useful indicator of neuromuscular fatigue (NMF) in elite footballers (Rowell et al., 2017). This CMJ metric had a dose–response relationship to match external load in various positional groups (Rowell et al., 2017). Although this finding supports the efficacy of using FT:CT to assess the post-match response, it is unknown whether pre-existing NMF measured via FT:CT impacts subsequent match performance in elite football as it does in other sports (Cormack et al., 2013; Mooney et al., 2013).

In team sport environments, monitoring training and competition load is aimed at maximizing performance (Gastin et al., 2013). However, there is limited understanding of the impact of previous training and competition load on subsequent match performance in elite football players. Furthermore, the influence of NMF, testosterone and cortisol on match performance is also unclear. Therefore, the purpose of this study was to examine the interactions between EWMA internal load of different time constants, NMF, hormonal response and match performance (measured via Coaches votes) (Cormack et al., 2013; Mooney et al., 2013) during a season of elite A-League football.

## Materials and Methods

Data for this study were collected from a single elite football team throughout a competitive season that included 34 matches (27 regular A-League, and seven Asian Champions league matches). A total of 23 players (excluding goal keepers) with a mean ± standard deviation (SD) age; 23.3 ± 4.1 years, height; 180 ± 10.0 cm and mass 75.7 ± 4.4 kg provided data for analysis. Given the tactical formation of the team was 4–5–1, players were parsed according to the positional groups of: center defender, wide defender, center midfielder, wide midfielder and striker in each game. The Victoria University Human Research Ethics Committee granted approval for this study with written informed consent obtained prior to commencement.

Given FIFA restrictions at the time of the study for the use of tracking devices during match play, sRPE was used to represent training and match loads. Athletes provided an RPE value 30 min post-training and match play (Borg et al., 1987), which was then multiplied by the total session duration to provide internal load (Foster, 1998; Impellizzeri et al., 2004).

Cumulative internal load was derived via EWMA (smoothed load). This approach uses a decay factor λ (lambda; value between 0 and 1), accounting for the decaying nature of load by assigning a higher weighting factor to more recent sessions (Hunter, 1986). The cumulative load was calculated by λ × (the previous day’s internal load) + (1 – λ) × (the cumulative internal load up to that point). The resulting cumulative load is effectively smoothed with the time constant given by the ratio 1/λ (λ = 1 over the number of days) (Lazarus et al., 2017). The smoothed load for this study was generated with λ of 0.33, 0.14, 0.1, 0.07, and 0.04 (representing time constants of 3-, 7-, 10-, 14-, and 28-day; day periods, respectively).

Coach rating of performance was used to determine individual athletes match performance, due to being previously established as a reliable measure from team sport coaches. Rating of match performance was collected from coaching and fitness staff (one head coach, two assistant coaches, one goal keeping coach, and one high performance manager) following match play. A Likert scale (1 = poor through to 5 = excellent) was used to rate each players’ performance in fulfilling their assigned role throughout the match (Cormack et al., 2008b; McLean et al., 2010). The mean of the 5 ratings was assigned to each player’s individual match performance.

Athlete’s CMJ and saliva collection were conducted before the last training session prior to match play (match day −1). Athletes were familiarized with the CMJ and saliva collection procedures prior to data collection. One maximal CMJ was performed on a force plate (400 Series Platform Plate; Fitness Technology, Adelaide, Australia) connected to manufacturer-supplied software (Ballistic Measurement System; Fitness Technology, Adelaide, Australia) following a standardized warm up and according to established protocols (Cormack et al., 2008a). The ratio of flight time to contraction time (FT:CT) is the measure most sensitive to variations in load in this cohort, and was therefore used for analysis (Cormack et al., 2008c; Rowell et al., 2017). Athletes provided 2 mL of saliva using the unstimulated passive drool technique between 09:00 h and 09:30 h following strict pre-test procedures (Cormack et al., 2008a). Samples were immediately frozen for later analysis. Duplicate enzyme-linked immunosorbent assay (Salimetrics, State College, PA, United States) using a microplate reader (SpectraMax 190, Molecular Devices, San Jose, CA, United States) determined testosterone [pg.mL^−1^] and cortisol [μg.dL^−1^] concentration (Cormack et al., 2008a).

Data were analyzed using quadratic mixed models in the Statistical Analysis System (version 9.4, SAS Institute, Cary, NC, United States). The quadratic model allowed for a curvilinear effect of internal load on match performance (coach rating) and on each of the pre-match test measures (FT:CT ratio, testosterone, cortisol and testosterone:cortisol ratio). Similar models allowed for a curvilinear effect of each pre-match measure on match performance. Fixed effects in the models were the intercept, the predictor (internal load or test measure), and the square of the predictor, which collectively estimated the mean quadratic effect. The random effects were player identity (to estimate different between-player means across the season), the interaction of player identity with the predictor and its square (to estimate individual differences in the quadratic effect), and the residual error (within-player weekly match or test variability). Effects of illness were adjusted for by including a dummy variable representing whether the player reported illness on the day of assessment as a fixed effect and as a random effect interacted with player identity. Separate analyses were performed for each playing position.

The quadratic effect of each predictor was assessed by deriving a within-player SD of the predictor by taking the square root of the mean of the squares on each individual player’s SD (weighted by degrees of freedom). The effects of a change in the predictor from −1 SD up to the mean and from the mean to +1 SD above the mean were then derived (Lazarus et al., 2017).

Effects on coach rating were derived and reported in raw units. Effects on pre-match test measures were derived after log transformation then back-transformed and expressed in percent units. Effects were assessed using non-clinical magnitude-based inference (MBI) (Batterham and Hopkins, 2006). The uncertainty in the effect was expressed as 90% confidence limits and with likelihoods (%) that the true value of the effect represented substantial or trivial changes expressed as possibly (25–75%), likely (75–95%), very likely (95–99.5%) and most likely (>99.5%) (Hopkins et al., 2009). When the confidence interval (the lower to upper confidence limit) for a change included substantial positive and negative values, the effect was deemed unclear. Given the inflation of overall Type-I error arising from large number of effects presented in this study, only those effects clear with 99% confidence intervals (shown in bold in the tables) were regarded as definitive. The smallest standardized change was considered to be 0.2, with standardized effects classed as: small, 0.20–0.60; moderate, 0.60–1.20; large, 1.2–2.0; very large, 2.0–4.0; and extremely large, >4.0 (Hopkins et al., 2009).

## Results

**FIGURE 1:**
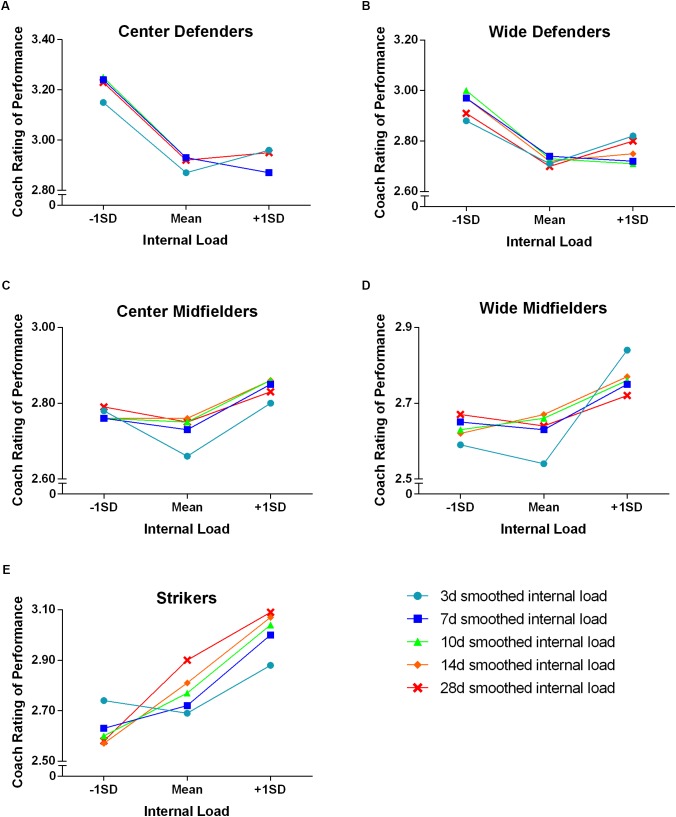
Quadratic analysis of the change in coach rating for a given change in internal load ±1 SD from the mean for **(A)** center defenders, **(B)** wide defenders, **(C)** center midfielders, **(D)** wide midfielders, and **(E)** strikers.

**FIGURE 2:**
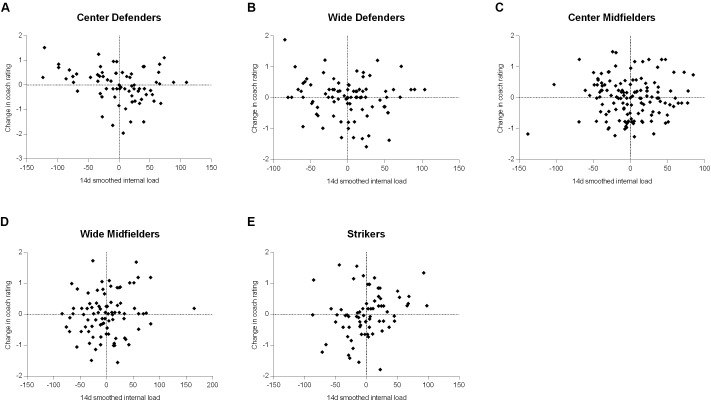
Scatter plot distribution of the change in coach rating for a given change in 14-day smoothed internal load for the different playing positions of **(A)** center defenders, **(B)** wide defenders, **(C)** center midfielders, **(D)** wide midfielders, and **(E)** strikers.

To provide a visual representation of the results of the quadratic analysis, **Figure 1** shows the change in coach rating for a 1 SD increase in internal load (from 1 SD below the mean to the mean and from the mean to 1 SD above the mean), across the EWMA time-periods for each playing position. Whilst a quadratic shape was fitted to the data to allow for non-linear relationships, the pure distribution of individual players’ change in coach rating for a given change in internal load is displayed in scatter plots in **Figure 2** (14-day EWMA smoothed load chosen for the example). There was little indication of individual differences in the quadratic relationships.

The impact of internal load on coach rating of performance is displayed in **Table 1**, with a number of substantial effects (small) observed. Center defenders showed a very likely reduction in coaches’ votes when 3- and 14-day smoothed load increased from −1 SD to the mean. Wide midfielders displayed a likely increase in coach rating when 3-day smoothed load increased from the mean to +1 SD above the mean. Similarly, strikers displayed a likely increased coach rating when smoothed load increased to +1 SD above the mean for the 7- and 10-day periods. There were numerous possible, trivial or unclear effects of an increase in internal load from the mean to ±1 SD above, particularly for center and wide defenders.

**Table 1:** Mean internal load across each smoothed time window and the corresponding coach rating in each positional group.

	Internal load (mean ± within-subject SD)	Coach rating at (mean ± between-subject SD)	Change in coach rating −1 SD to mean (mean, ±90%CL)	Change in coach rating mean to +1 SD (mean, ±90%CL)
**3-Day smoothed internal load**			
Central defender	246 ± 58	2.87 ± 0.65	**-0.28±0.14^∗∗∗^**	0.09±0.22*
Wide defender	235 ± 73	2.71 ± 0.66	-0.18±0.26*	0.11±0.26
Central midfield	232 ± 52	2.66 ± 0.64	-0.11±0.12*	0.14±0.12*
Wide midfield	251 ± 59	2.54 ± 0.77	-0.05±0.15^00^	**0.29±0.20^∗∗^**
Striker	233 ± 66	2.69 ± 0.76	-0.05±0.15^00^	0.18±0.23*
**7-Day smoothed internal load**						
Center defender	246 ± 52	2.93 ± 0.64	-0.30±0.41**	-0.06±0.30
Wide defender	240 ± 51	2.74 ± 0.62	-0.24±0.29*	-0.02±0.26
Central midfield	237 ± 41	2.73 ± 0.66	-0.03±0.10^000^	0.13±0.12*
Wide midfield	254 ± 46	2.63 ± 0.79	-0.02±0.20	0.12±0.15*
Striker	237 ± 47	2.72 ± 0.75	0.09±0.15*	**0.28±0.21^∗∗^**
**10-Day smoothed internal load**						
Center defender	247 ± 51	2.93 ± 0.64	-0.32±0.34**	-0.06±0.30
Wide defender	244 ± 46	2.73 ± 0.62	-0.26±0.30**	-0.02±0.27
Central midfield	239 ± 39	2.75 ± 0.66	-0.01±0.10^0000^	0.12±0.11*
Wide midfield	257 ± 44	2.66 ± 0.79	0.03±0.20	0.10±0.14*
Striker	240 ± 42	2.77 ± 0.73	0.17±0.27*	**0.28±0.23^∗∗^**
**14-Day smoothed internal load**
Center defender	249 ± 51	2.93 ± 0.65	**-0.31±0.15^∗∗∗^**	-0.06±0.18^00^
Wide defender	248 ± 42	2.72 ± 0.62	-0.25±0.27**	0.03±0.25
Central midfield	242 ± 38	2.76 ± 0.66	-0.00±0.16	0.11±0.24*
Wide midfield	259 ± 44	2.67 ± 0.79	0.05±0.21	0.10±0.14*
Striker	244 ± 39	2.81 ± 0.72	0.24±0.24**	0.25±0.24**
**28-Day smoothed internal load**						
Center defender	255 ± 52	2.92 ± 0.66	-0.31±0.25**	0.03±0.36
Wide defender	257 ± 33	2.70 ± 0.63	-0.21±0.22*	0.10±0.22*
Central midfield	248 ± 35	2.75 ± 0.66	-0.04±0.11^00^	0.08±0.10^00^
Wide midfield	266 ± 44	2.64 ± 0.78	-0.03±0.23	0.08±0.14^00^
Striker	251 ± 33	2.90 ± 0.71	**0.32±0.25^∗∗^**	0.19±0.24*

*The raw change in coach rating for a given increase in internal load from −1 SD up to the mean and from the mean to +1 SD above the mean is also shown.*

*90%CL, 90% confidence limits.*

*Values in bold represent substantial effects clear at the 99% level. Symbols denote: ^∗^possibly, ^∗∗^likely, ^∗∗∗^very likely, and ^∗∗∗∗^most likely chance of the true effect being a substantial change (standardized effect > 0.20). Trivial effects are classified: ^0^possibly, ^00^likely, ^000^very likely, and ^0000^most likely. Unclear effects do not have a symbol.*

Similarly, there were a large number of unclear interactions between the change in pre-match FT:CT, testosterone, cortisol, and testosterone:cortisol and rating of match performance (**Table 2**). The only clear (small) effect was observed in the wide midfielders, where an increase in testosterone from −1 SD to the mean resulted in a likely increase in coach rating.

**Table 2:** Mean coach rating of performance and the raw change in coach rating for a given increase in season average FT:CT, cortisol, testosterone and testosterone:cortisol from −1 SD to the mean and from the mean to +1 SD above the mean is displayed relative to positional group.

	Coach rating at mean (mean ± between-) subject SD)	Change in coach rating −1 SD to mean (mean, ±90%CL)	Change in coach rating mean to +1 SD (mean, ±90%CL)
**Change in performance for a given value of FT:CT relative to mean**						
Center defender	2.98 ± 0.74	-0.28 ± 0.36**	-0.25 ± 0.31**
Wide defender	2.67 ± 0.54	-0.06 ± 0.29	0.05 ± 0.39
Central midfield	2.93 ± 0.52	0.01 ± 0.14	-0.07 ± 0.11^00^
Wide midfield	2.42 ± 0.60	-0.25 ± 0.23**	-0.08 ± 0.24
Striker	3.12 ± 0.80	0.11 ± 0.57	0.01 ± 0.82
**Change in performance for a given value of cortisol relative to mean**						
Center defender	2.92 ± 0.75	-0.12 ± 0.25	-0.20 ± 0.34
Wide defender	3.08 ± 0.47	0.12 ± 0.43	-0.10 ± 0.42
Central midfield	2.97 ± 0.55	-0.04 ± 0.25	-0.09 ± 0.27
Wide midfield	2.91 ± 0.67	0.24 ± 0.44	0.01 ± 0.46
Striker	3.04 ± 0.61	0.06 ± 0.30	0.03 ± 0.30
**Change in performance for a given value of testosterone relative to mean**						
Center defender	2.96 ± 0.75	0.03 ± 0.24	-0.00 ± 0.23
Wide defender	3.09 ± 0.51	0.04 ± 0.31	-0.07 ± 0.34
Central midfield	2.86 ± 0.54	0.04 ± 0.18	0.03 ± 0.19
Wide midfield	2.98 ± 0.70	**0.29±0.22^∗∗^**	0.11±0.13*
Striker	3.00 ± 0.64	0.14 ± 0.27	0.16 ± 0.64
**Change in performance for a given value of T:C relative to mean**						
Center defender	2.93 ± 0.71	0.25 ± 0.31**	0.12 ± 0.26
Wide defender	3.11 ± 0.46	0.11 ± 0.25	-0.14 ± 0.17*
Central midfield	2.99 ± 0.58	0.13 ± 0.15*	-0.00 ± 0.12
Wide midfield	3.04 ± 0.61	0.18 ± 0.40	-0.18 ± 0.39
Striker	3.02 ± 0.55	0.11 ± 0.34	0.12 ± 0.27

*90%CL: 90% confidence limits.*

*Values in bold represent the substantial effects that are clear at the 99% CI. Symbols denote: ^∗^possibly, ^∗∗^likely, ^∗∗∗^very likely, and ^∗∗∗∗^most likely chance that the true effect is a substantial change (standardized effect > 0.20). Trivial effects are classified: ^0^possibly, ^00^likely, ^000^very likely, and ^0000^most likely. Unclear effects do not have a symbol.*

The impact of smoothed load on FT:CT was mostly unclear or trivial. However, a small effect was observed in the center defenders who showed a likely higher FT:CT when 14-day smoothed load increased from the mean to +1 SD above the mean. In contrast to the limited impact on FT:CT, cortisol was substantially influenced by internal load. Center midfielders had a very likely increased cortisol when 7- and 10-day smoothed load increased from −1 SD to the mean (small effects). Center defenders had a most likely increase in cortisol when 3-day smoothed load increased from the mean to +1 SD above the mean (large effect). Further, increased 14- and 28-day smoothed load from −1 SD to the mean and from the mean to +1 SD above the mean resulted in increased cortisol (small and moderate effects, respectively). Wide defenders cortisol likely increased when 10- and 14-day smoothed load increased from the mean to +1 SD above the mean (small effects). Wide midfielders also had a very likely increased cortisol with an increase in 28-day smoothed load from −1 SD to the mean (small effect).

Center defenders had a very likely (moderate effect) and likely (small effect) increase in testosterone when 3- and 28-day smoothed load increased from the mean to +1 SD above the mean, respectively. Wide midfielders also had a very likely and likely increased testosterone (small effects) when 28-day smoothed load increased from −1 SD to the mean and from the mean to +1 SD above the mean, respectively.

Increased internal load resulted in several substantial reductions in testosterone:cortisol. Specifically, center defenders had a very likely reduction when 3- and 7-day smoothed load increased from the mean to +1 SD above the mean (large effects). Wide defenders had the same response with a likely reduction in testosterone:cortisol when 10- and 14-day smoothed load increased from the mean to +1 SD above the mean (small effects). Center midfielders also had a reduced testosterone:cortisol when 7-, 10-, and 14-day smoothed load increased from −1 SD to the mean (small effects).

## Discussion

The quadratic analysis utilized in this study revealed that across playing positions, mean cumulative internal load did not produce the best match performance. Furthermore the relationship between internal load and match performance was impacted by the length of the analysis window with 3- to 14-day periods most influential. This finding indicates that recent load is relatively more important to the performance of high level football players than load accumulated over longer periods. Dividing the sample into playing positions resulting in a small *n* for each position combined with minimal variation in training load between individuals within a position, resulted in a limited ability to estimate individual responses.

### Interaction Between Internal Load and Match Performance

Center defenders had a substantial reduction in coach rating when internal load increased from −1 SD below to the mean in the 3- and 14-day windows. Although center defenders cover the lowest relative distances (m.min^−1^) and also have the lowest internal load compared to the other playing positions during matches (Torreño et al., 2016), a large proportion of match load in this group is accumulated through body contacts and other movements apart from running (Bloomfield et al., 2007; Dellal et al., 2010; Arrones et al., 2014; Torreño et al., 2016). These movements (that are independent of running) potentially induce high levels of muscle damage and/or NMF, resulting in the need for a longer recovery time (Bloomfield et al., 2007; Johnston et al., 2014). As a result of the greater non-running stress, players in this positional group may benefit from a reduced training load in order to allow them to recover from each match. Furthermore, given their tactical role and associated importance of maintaining defensive structure and organization to prevent the opposition forward from scoring, center defenders may have a higher psychological load compared to the other positions (Dellal et al., 2010). This psychological load is likely to be a substantial contributing factor to their RPE (Blanchfield et al., 2014; Gallo et al., 2015) and further supports the suggestion that this position is likely to benefit from relatively lower training loads in the immediate pre-match period. Whist reductions in coach rating were found with increases in load across all EWMA periods, the biggest effects occurred in the 3- and 14-day windows (-0.28 ± 0.14 and -0.31 ± 0.15). This suggests that planning the training load of center defenders may be best achieved by manipulating 3- and 14-day periods and ensuring their load is not greater than −1 SD below the mean. Wide defenders displayed a similar pattern of lower match performance from increasing internal load and may be the result of similar match activity profiles to those of center defenders (Di Salvo et al., 2007; Torreño et al., 2016). Due to their tactical requirements in match play, wide defenders’ biggest contribution of load, however, occurs through repeat high-intensity efforts and sprints as they provide an overload wide passing option (Arrones et al., 2014; Torreño et al., 2016). The findings for the wide defenders suggests a training load monitoring and manipulation approach similar to that adopted for center defenders (i.e., lower training loads) may be appropriate.

In contrast to defenders, wide midfielders and strikers displayed increased coach ratings when internal load was +1 SD above the mean. The largest effects occurred across the more acute windows of 3-day EWMA for wide midfielders, and 7- and 10-day EWMA periods for the strikers. It appears, like the defenders, there is an incongruity between mean training load and match performance. As midfielders generally produce the highest match activity profiles (Di Salvo et al., 2007; Arrones et al., 2014; Torreño et al., 2016), the mean training load in this study may have been an insufficient stimulus to prepare this positional group for match play. Despite the relationships between internal load and performance being seemingly opposite in defenders compared to midfielders, it appears that monitoring training load should occur across a similarly short window. In the case of midfielders, optimizing training may be best achieved by using a window of 3–10 days. It appears that more chronic training load (i.e., longer than 10 days) is relatively unimportant in acute match performance, however, there may be interactions with other aspects such as injury (Hulin et al., 2016; Malone et al., 2016).

### Impact of FT:CT and Testosterone, Cortisol, and Testosterone:Cortisol on Coach Rating of Performance

The ratio of FT:CT provides a useful marker of altered movement strategy as a result of NMF (Cormack et al., 2008a; Rowell et al., 2017). A reduction in pre-match FT:CT of >8% moderated Australian rules football players movement efficiency in subsequent match play and resulted in a lower coach rating of performance (Cormack et al., 2013; Mooney et al., 2013). Furthermore, a reduction in FT:CT post-match compared to baseline was also negatively correlated with subsequent match performance (Cormack et al., 2008b). However, despite a moderate to high accumulated PlayerLoad^TM^ (>500 au) during A-League match play being shown to cause suppressed FT:CT for 42 h (Rowell et al., 2017), in the current study many of the associations between FT:CT and coach rating of subsequent performance were unclear. Whilst FT:CT provides a useful recovery measure in this population (Rowell et al., 2017), it did not display a clear impact on performance as measured by coach rating in this study. The current findings may be a function of a shorter neuromuscular recovery in A-League players (42 h) (Rowell et al., 2017) compared to Australian Rules (72 h) (Cormack et al., 2008a). Due to this, although players may have been classed as “fatigued” based on their FT:CT on match day −1, they may have been nearly fully recovered by match time (∼36 h post FT:CT assessment). As a result, the FT:CT value on match day −1 may not have been a true reflection of suppressed neuromuscular function during the match. Although FT:CT assessed on match day −1 may not be directly related to coach rating of performance in football players, it may act as a moderator of movement strategy (Cormack et al., 2013; Mooney et al., 2013), however, this is yet to be explored in football players.

Higher pre-match testosterone has been associated with improved match performance in other football codes such as Australian Rules and rugby (Cormack et al., 2008b; Cook and Crewther, 2012). Similarly, wide midfielders in the current study had an increased rating of match performance when testosterone increased from −1 SD to the mean; however, no clear interaction was evident in the other positions. Increased testosterone may also reflect regeneration and recovery from previous load (Carré et al., 2006; Crewther et al., 2013). Heightened testosterone pre-match is associated with preparatory, aggressive and dominant behavior increasing assertiveness and vigor leading to a dominant and winning performance (Kraemer et al., 2004; Argus et al., 2009; Martínez et al., 2010; Crewther et al., 2013; Gaviglio et al., 2014). The elevation of testosterone that occurred in the wide midfielders when internal load was above the mean may have improved performance through similar mechanisms. It appears that a higher training load in this positional group may provide an anabolic stimulus that subsequently impacts performance (Meckel et al., 2011; Kilian et al., 2016). In contrast, the lack of interaction with performance appears to somewhat limit the usefulness of both cortisol and testosterone:cortisol for regular performance monitoring in A-League football, however, substantial relationships with previous training load may suggest some benefit for their use (see below).

### Impact of Internal Load on FT:CT and Hormonal Response

Whilst external load impacts the post-exercise response of various performance and biochemical markers (Cormack et al., 2008a; Rowell et al., 2017; Tofari et al., 2017), prior internal load has also demonstrated an influence. For example, the increase in internal load (RPE-based) from basketball match play was closely correlated to an increase in post-match cortisol response (*r* = 0.75) (Moreira et al., 2012).

In the current study, the interaction between internal load and FT:CT was predominantly unclear. This was similar to the impact of the change in FT:CT on performance. However, a clear effect was evident in center defenders, where an increase in internal load by +1 SD above the mean 14-day EWMA resulted in a likely increase in FT:CT. Paradoxically, increased internal load in the same positional group and time period was associated with lower performance ratings. Whilst it appears that an elevated training load provides some kind of stimulating effect on jumping performance, it also negatively impacts coach perceptions of performance. The exact mechanism at play here is unclear and requires further investigation.

Testosterone, is an anabolic hormone that has been shown to increase across a competitive Australian Rules football season (Cormack et al., 2008b). Such a response suggests team sport players may be able to maintain an anabolic hormonal environment even during long competitive seasons. Similarly, testosterone increased in response to a single match in A-League football players (Rowell et al., 2017) and the results of the current study are in agreement for wide midfielders and center defenders with an increased testosterone over the 28-day EWMA window. The increase in testosterone that occurs when training load increases +1 SD above the 28-day mean suggests that a relatively high chronic training load plays a role in creating an anabolic environment in these athletes (Kraemer et al., 2004; Cormack et al., 2008b). Similarly, the increase in testosterone in center defenders as a result of elevated 3-day internal load suggests that the acute training stimulus also creates such an anabolic stimulus (Urhausen et al., 1995; Eliakim et al., 2009). As a result, it appears that increasing the training load above the mean for center defenders in particular, has a beneficial effect on hormones, however, this does not coincide with performance improvements.

A change in internal load from −1 SD below to the mean and from the mean to +1 SD above was associated with increased cortisol in wide midfielders and both defensive groups across various EWMA windows. This finding is similar to previous work in professional basketball players, where heightened cortisol was related to session RPE (*r* = 0.75) (Moreira et al., 2012). Cortisol is a stress hormone, that has increased in response to training and match load in team sport athletes (Filaire et al., 2003; Rowell et al., 2017). In the case of center defenders, increasing internal load appears to result in both an increase in cortisol and compromised performance. This may be a function of both the physiological and psychological stress associated with playing and training for defensive players and further supports the notion that a relatively lower training load would be beneficial.

A decreased testosterone:cortisol ratio, particularly in excess of 30% is suggested to be reflective of a catabolic state (Viru and Viru, 2004). A-League match play results in a reduced testosterone:cortisol (Rowell et al., 2017) and the current findings suggest increased training loads above the mean substantially reduce testosterone:cortisol, particularly in defensive players. Examination of the individual testosterone and cortisol response suggests in many cases there has been an increase in testosterone, however, this can be outweighed by an increase in cortisol and result in a decreased testosterone:cortisol (Hayes et al., 2015). Furthermore, as mentioned above, the lack of association between testosterone:cortisol and performance in the current study casts doubt on the suitability of testosterone:cortisol as a regular monitoring tool for associated match performance in A-League Football players.

## Conclusion and Practical Applications

The impact of training load on performance in A-League players appears to be position specific. Wide-midfielders and strikers performance is enhanced with training load above the mean, whilst central defenders appear to benefit from a relatively lower training load. It appears in this population that relatively recent training load (i.e., 3–14 days) is more important to match performance than load accumulated over longer periods.

The hormonal response to training appears to be impacted by both very short (3-day) and relatively long (28-day) preceding workloads. Both these periods stimulate an increase in testosterone, although this was only associated with improved performance in wide midfielders. Furthermore, there are a large number of unclear effects on FT:CT as a result of changes in training load. A similar pattern exists in relation to changes in performance due to changes in FT:CT and hormonal variables. A limitation of this study was the inability to assess the impact of velocity and accelerometer derived external load metrics on FT:CT, hormonal response and match performance, due to restrictions on wearable devices during match play. Whilst several unclear changes were identified between RPE based internal load, FT:CT and hormonal response, further research is warranted to identify the impact of external load metrics. Based on the current results the use of testosterone, cortisol and FT:CT as indicators of subsequent performance is questionable, however, this may be impacted by the timing of testing relative to match play. Furthermore, the division of the sample into playing positions resulting in a small n for each position combined with minimal variation in training load between individuals within a position may result in a limited ability to estimate individual responses. Whilst the exact mechanism at play here is unclear and requires further investigation, it is possible that FT:CT may be more sensitive to external load (e.g., accelerometer and GPS derived metrics) than RPE based internal load. It therefore appears that monitoring FT:CT and hormonal markers in the applied setting is important to consider timing of testing, and the sensitivity to both internal and external load metrics within specific athlete populations.

The application of the results of this research do, however, suggest that improving performance in A-League players, can be assisted by the manipulation of training load on a position-specific basis. Specifically, coaches and support staff could maximize the performance of strikers by providing a workload above the 7- and 10-day mean internal load. Similarly, the performance of wide midfielders may be enhanced by increasing load above the 3-day smoothed mean. Conversely, center defenders training loads might be reduced below their respective mean internal loads for the relevant length windows. In order to achieve this, coaches and support staff should monitor internal training load via the use of session RPE and calculate the smoothed load using an EWMA for relatively short time constants (i.e., 3–14 days). Subsequent training can then be adjusted to achieve the desired internal load.

## Author Contributions

AR, SC, and RA: conceived and designed the study, interpreted the results, drafted the manuscript, and prepared tables and figures. AR: data collection and analysis. AR, WH, SC, BL, and AE: statistical data analysis and interpretation. AR, SC, RA, WH, BL, AE, and AS: edited, critically revised the manuscript, and approved the final version.

## Conflict of Interest Statement

The authors declare that the research was conducted in the absence of any commercial or financial relationships that could be construed as a potential conflict of interest.
